# Measles outbreak amplified in a pediatric ward: Lyantonde District, Uganda, August 2017

**DOI:** 10.1186/s12879-020-05120-5

**Published:** 2020-06-05

**Authors:** Claire Biribawa, Joselyn Annet Atuhairwe, Lilian Bulage, Denis Othuba Okethwangu, Benon Kwesiga, Alex Riolexus Ario, Bao-Ping Zhu

**Affiliations:** 1 Uganda Public Health Fellowship Program, Kampala, Uganda; 2 Ministry of Health, Kampala, Uganda; 3 US Centers for Disease Control and Prevention, Kampala, Uganda; 4 Division of Global Health Protection, Center for Global Health, US Centers for Disease Control and Prevention, Atlanta, USA

**Keywords:** Pediatric measles outbreak, Nosocomial infection, Global health security, Vaccine-effectiveness, Vaccination-coverage

## Abstract

**Background:**

Measles is a highly infectious viral disease. In August 2017, Lyantonde District, Uganda reported a measles outbreak to Uganda Ministry of Health. We investigated the outbreak to assess the scope, factors facilitating transmission, and recommend control measures.

**Methods:**

We defined a probable case as sudden onset of fever and generalized rash in a resident of Lyantonde, Lwengo, or Rakai Districts from 1 June-30 September 2017, plus ≥1 of the following: coryza, conjunctivitis, or cough. A confirmed case was a probable case with serum positivity of measles-specific IgM. We conducted a neighborhood- and age-matched case-control study to identified exposure factors, and used conditional logistic regression to analyze the data. We estimated vaccine effectiveness and vaccination coverage.

**Results:**

We identified 81 cases (75 probable, 6 confirmed); 4 patients (4.9%) died. In the case-control study, 47% of case-patients and 2.3% of controls were hospitalized at Lyantonde Hospital pediatric department for non-measles conditions 7–21 days before case-patient’s onset (OR_adj_ = 34, 95%CI: 5.1–225). Estimated vaccine effectiveness was 95% (95%CI: 75–99%) and vaccination coverage was 76% (95%CI: 68–82%). During the outbreak, an “isolation” ward was established inside the general pediatric ward where there was mixing of both measles and non-measles patients.

**Conclusions:**

This outbreak was amplified by nosocomial transmission and facilitated by low vaccination coverage. We recommended moving the isolation ward outside of the building, supplemental vaccination, and vaccinating pediatric patients during measles outbreaks.

## Background

Measles is an acute viral infectious disease causing approximately 45 million infections and 1 million deaths annually worldwide, mostly in children [1–3]. In Africa, an estimated 13 million infections and nearly 650,000 deaths occur annually, with sub-Saharan Africa having the highest morbidity and mortality [4].

Measles is transmitted via droplets from the nose, mouth, or throat of infected persons. Initial symptoms may include high fever, runny nose, conjunctivitis, and Koplik spots, which usually appear 10–12 days after infection. A rash develops 14 (range: 7–21) days after exposure, starting on the face and upper neck and gradually spreading downwards. Patients are infectious starting approximately four days before to four days after rash onset [5].

Treatment for measles virus infection is only supportive and most patients recover within 2 to 3 weeks. However, measles can cause serious complications, including blindness, encephalitis, severe diarrhea, ear infection, and pneumonia, particularly in malnourished children and immune-compromised patients [5–7]. The case-fatality rate in developing countries is usually 3–5%; however, in some localities it may be as high as 10–30% [5, 8].

Measles vaccination is the best strategy for preventing measles outbreaks and achieving the goal of measles elimination. To effectively prevent measles outbreaks and achieve the goal of measles elimination, WHO recommends a 2-dose vaccination administered at 9 and 15–18 months of age, and a Vaccination Coverage (VC) at ≥80% for the 2-dose vaccination schedule [9]. The Uganda National Expanded Programme on Immunization, as in most countries in the WHO-AFRO region, currently implements a one-dose vaccination at 9 months of age. However Supplemental Immunization Activities are organized periodically to interrupt transmission and spread.

On 3 August 2017, the Uganda Ministry of Health (UMoH) received a notification of a measles outbreak in Lyantonde District. Serum samples from six suspected measles patients tested positive for measles-specific IgM at the Uganda Virus Research Institute. To support the district in controlling this outbreak, we conducted an epidemiological investigation to determine the scope of the outbreak, assess risk factors for transmission, assess vaccine effectiveness and recommended evidence-based measures to prevent future outbreaks.

## Methods

### Study setting

The outbreak occurred in a tri-district area in Central Uganda comprising Lyantonde, Lwengo, and Rakai districts. The estimated total population was approximately 926,000 (101,200 in Lyantonde, 281,400 in Lwengo, and 543,400 in Rakai), based on the 2017 projected populations from the 2014 census [10]. The three districts border each other and share several public hospitals in Lyantonde District.

### Case definition and case finding

We defined a probable case as sudden onset of fever and generalized rash in a resident of the tri-district area from 1 June to 30 September 2017, plus ≥1 of the following: coryza, conjunctivitis, or cough. A confirmed case was a probable case with measles-specific IgM positivity.

For case-finding, we reviewed outpatient and inpatient records at health facilities in the tri-district area, and actively searched for cases with the help of members of village health teams and community leaders. We collected data on patients’ symptoms, onset dates of symptoms, treatment outcomes, demographic characteristics, place of residence, receipt of care, and vaccination status.

### Descriptive epidemiology

We analyzed the line-listed cases by onset of symptoms, age, sex, and place of residence. To calculate attack rates (AR) by age and sex, we used the estimated population in the tri-district area, provided by Uganda Bureau of Statistics [9]. We drew a choropleth map using QGIS software to describe the ARs by sub-county for Lyantonde District, where most (68%) of the cases came from.

### Hypothesis generation

Using a semi-structured questionnaire, we interviewed a convenience sample of 15 caretakers for case-patients regarding their potential exposures during their likely exposure period (i.e., 7–21 days before their rash onset, or between minimum and maximum incubation periods). The exposures of interest included attending social gatherings, attending worship places, visiting health facilities, visiting communal gathering points, and immunization status. We generated hypotheses about exposures based on findings from the descriptive epidemiology analysis and hypothesis-generation interviews.

### Case-control study

We conducted a case-control study to test the hypothesis on potential exposures. At the time of the case-control study, 38 case-patients were line-listed. We recruited 34 of those case-patients aged ≥1 year to participate in the case-control study. For each case, we selected 4 controls in the same immediate neighborhood (i.e., within three homes of the case-patient’s) who had no measles symptoms from 1 July to 30 September 2017. We individually matched controls to the case by age (±2 years). We assessed the exposure risk factors for both the case-patient and the matched controls during the case-patient’s likely exposure period, using a structured questionnaire. Vaccination status was determined by either reviewing the vaccination records or, if unavailable, by asking whether the child had received an injection on the upper arm at 9 months of age (which is the standard practice for measles vaccine in Uganda). Cases and controls were considered vaccinated only if they were vaccinated prior to the onset of the outbreak. We also collected data on demographic characteristics (e.g., age and sex) of both case-patients and controls.

To account for individual matching in the study design, we used conditional logistic regression to analyze the data, using the matched set as the matching variable. We first assessed the association between each individual risk factor and measles. Risk factors that were statistically significant at the *p* < 0.05 level during the univariate analysis were included in the multivariate conditional logistic regression model to calculate the adjusted odds ratios (OR_adj_) and their associated 95% confidence intervals (CI). Non-significant variables in the multivariable model (*p* ≥ 0.05) were backward-eliminated until all were significant. Multivariate logistic regression analysis was used to control for potential confounding variables that could be included in the model by holding all the other variables constant.

### Estimation of vaccine effectiveness (VE) and vaccination coverage (VC)

We estimated measles VE using the following formula [11]:

$$ \mathrm{VE}=1-{\mathrm{OR}}_{\mathrm{adj}} $$

where OR_adj_ is the odds ratio associated with having been vaccinated for at least one dose of measles vaccine, adjusted for risk factors that were significant during the univariate analysis, using conditional logistic regression.

We estimated the VC using the percentage of controls with a history of measles vaccination in the case-control study, assuming the controls to be representative of the general population. We also obtained the administrative data from the district surveillance officer on VC in Lyantonde District.

### Environmental assessment

We observed the layout of the pediatric department at Lyantonde Hospital, especially the pediatric wards in question, and examined the ventilation system. We also reviewed the patient log to assess the type of illnesses admitted in the pediatric department.

## Results

### Descriptive epidemiology

Between 1 June and 30 September 2017, we found 81 cases (75 probable and 6 confirmed) in the tri-district area, with four deaths (case fatality rate = 4.9%). Of these cases, 55 were from Lyantonde, 16 from Rakai, and 10 from Lwengo District. Common symptoms included fever (100%), rash (100%), coryza (96%), cough (92%), and conjunctivitis (91%).

Among those that died, there was one female and 3 males, aged 11–17 months. Only one case that died had history of one dose measles vaccination. The major cause of death was respiratory complications (3/4) and the cause of death could not be ascertained for one of the cases. All the cases that died were managed at home for the measles virus infection. In addition 3 of those cases had other underlying disease conditions.

**Fig. 1:**
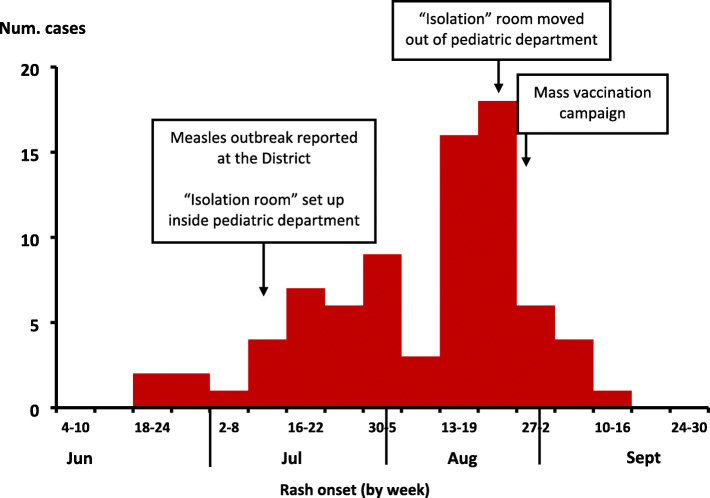
Epidemic curve of 79 measles cases showing the number of cases by week of fever onset during an outbreak: Lyantonde, Lwengo and Rakai districts, Uganda, June–September 2017

The initial cases had rash onset on 21 June. Cases started to increase in July and August, and the last case occurred on 12 September. The epidemic curve was indicative of a propagated outbreak. An emergency mass vaccination was rolled out in the tri-district area. This involved health facility based mass vaccination campaign for all children below 5 years of age (Fig. 1).

Of all age groups, children aged 9 months–5 years (AR = 32/100,000) and those 4–9 months (AR = 17/100,000) were the most affected. Females (AR = 9.4/100,000) and males (AR = 8.1/100,000) were similarly affected (Table 1).

**Table 1:** Measles attack rate by age and sex during an outbreak: Lyantonde, Lwengo and Rakai districts, June–September 2017

Characteristics	Population	Num. of cases	AR (/100,000)
Overall (three districts)	926,000	81	8.7
District			
Lyantonde	101,200	55	54
Rakai	543,400	16	2.9
Lwengo	281,400	10	3.6
Age group			
< 9 m	35,610	6	17
9 m–5y	158,320	50	32
6–18y	336,930	21	06.2
≥ 18y	395,140	4	1.01
Sex			
Male	457,100	37	8.1
Female	468,900	44	9.4

**Fig. 2:**
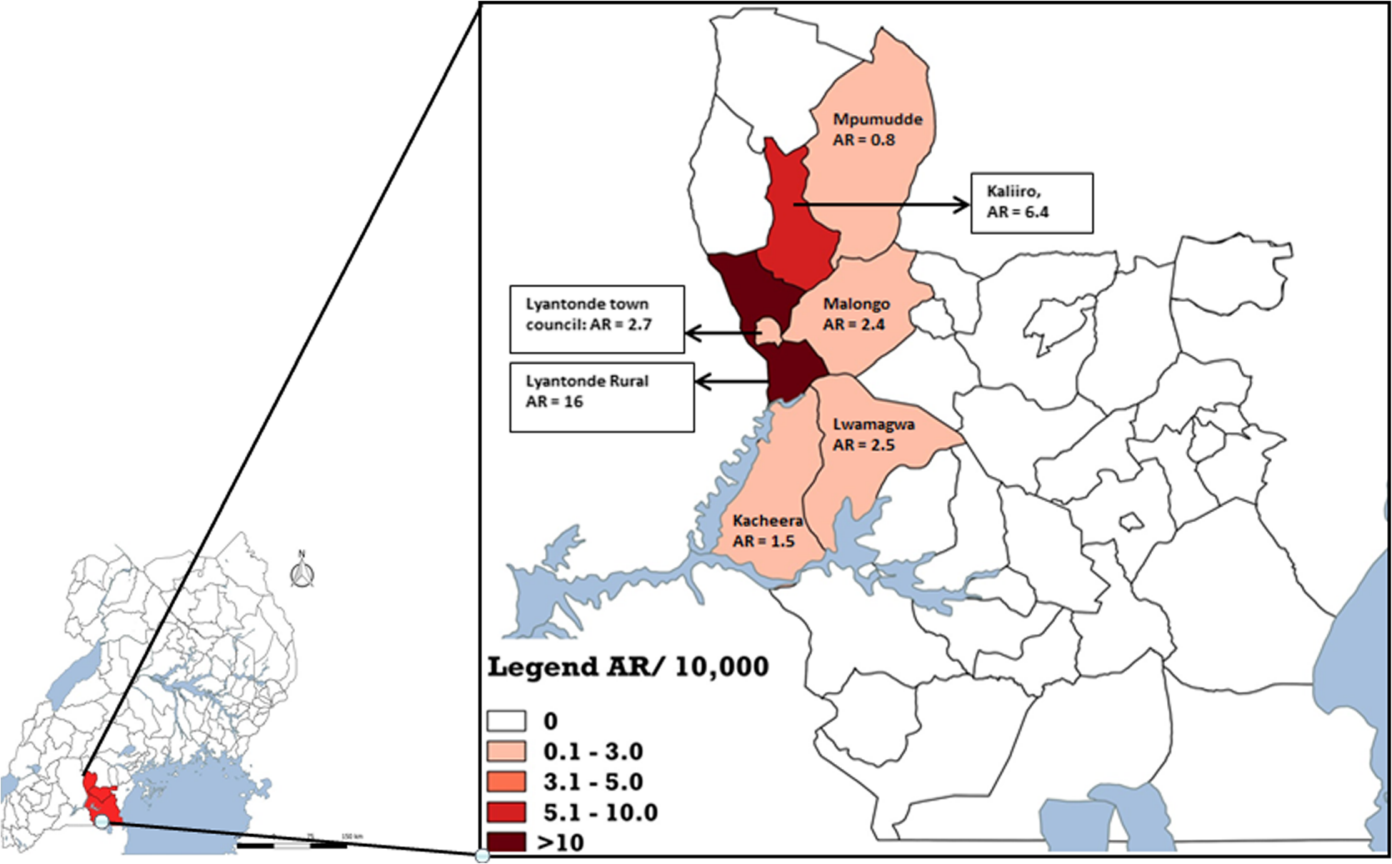
Attack rate (per 10,000) by sub-county during a measles outbreak: Lyantonde, Lwengo and Rakai districts, Uganda, June–September 2017

The attack rate in the tri-district area was 8.7/100,000. The initial cases in June occurred in Rakai District. The outbreak started to affect Lyantonde District in July, and later spread to villages in Lwengo District bordering Lyantonde District. Lyantonde District had the highest AR (54/100,000) of all 3 districts (with 68% of all cases). Within Lyantonde District, Lyantonde Rural Sub-county was the most affected (AR = 16/10,000) (Fig. 2).

### Findings from the hypothesis generation interviews

Of the 15 probable measles case-patients interviewed, 67% reported having visited Lyantonde Hospital during the 3 weeks before onset of symptoms; 40% of the patients reported having gone to school, 20% had visitors with measles at home, 20% went to a church, and 13% went to communal water-collection points; 73% of the case-patients had no history of measles vaccination.

### Case-control study findings, VC, and VE

In the case-control study, case-patients and controls were comparable in mean age (6.0 years among case-patients vs. 5.9 years among controls) and sex distribution (41% of case-patients and 42% of controls were males). During the bivariate analysis, 44% of case-patients and 2.3% of controls had been hospitalized in the pediatric department of Lyantonde Hospital for non-measles conditions 7–21 days before case-patients’ rash onset (OR = 30, 95% CI: 7.0–132). Visiting any health facility 7–12 days before case-patient’s rash onset was a significant risk factor. Going to church and going to communal water collection points were inversely associated with illness. In the final conditional logistic regression model, hospitalization at the pediatric department (OR_adj_ = 34, 95%CI: 5.1–225), going to communal water collection points (OR_adj_ = 0.056, 95%CI: 0.0066–0.47) and measles vaccination history (OR_adj_ = 0.051, 95%CI: 0.011–0.25) remained significant. All vaccinated cases and controls reported received at least one dose of measles vaccine. The associations of measles with other risk factors became non-significant (Table 2).

**Table 2:** Association between measles and exposures during an outbreak: Lyantonde, Lwengo and Rakai districts, Uganda, June–September 2017

Exposure^a^	% cases (*n* = 34)	% controls (*n* = 136)	OR ^b^ (95% CI)	OR_adj_^c^ (95% CI)
Exposures during case-patient’s likely exposure period^d^				
Hospitalized at pediatric department, Lyantonde Hospital	47	2.3	30 (7.0–132)	34 (5.1–225)
Visited any health facility	59	36	2.6 (1.2–5.5)	
Went to communal water point	12	39	0.14 (0.039–0.51)	0.056 (0.0066–0.47)
Went to church	38	61	0.36 (0.16–0.81)	
Went to school	47	41	1.4 (0.55–3.6)	
History of measles vaccination	26	76	0.11 (0.043–0.27)	0.051 (0.011–0.25)

^a^Some records had missing values for exposure variables, including 4 for “hospitalized at pediatric department, Lyantonde Hospital”, 1 for “went to communal water point”, 2 for “went to church”, and 2 for “went to school”. These records were excluded from the respective analysis

^b^OR = Crude odds ratios from univariate conditional logistic regression analysis, in which the matching variable was the case-control set

^c^OR_adj_ = Odds ratios from multivariable conditional logistic regression

^d^Case-patient’s likely exposure period = 7–21 days (minimum-to-maximum incubation periods) before case-patient’s rash onset

The estimated VE was 95% (95% CI: 75–99%). The estimated VC, based on the percent of controls that had a history of measles vaccination, was 76% overall, and did not differ greatly between age groups (Table 3). The estimated VC based on administrative data for Lyantonde District was 83%.

**Table 3:** Measles vaccination coverage by age during an outbreak: Lyantonde, Lwengo and Rakai districts, Uganda, June–September 2017

Age (years)	Vaccination Coverage (%)^a^	95% CI
Overall	76 (101/134)	68–82
9–12 months	86(12/14)	52–98
9 months-5y	72 (64/90)	62–80
6-18y	77(29/38)	60–89
>18y	83(5/6)	41–99

^a^Estimated based on the percent of controls in the case-control study who have been vaccinated

### Assessment of the pediatric department at Lyantonde Hospital

**Fig. 3:**
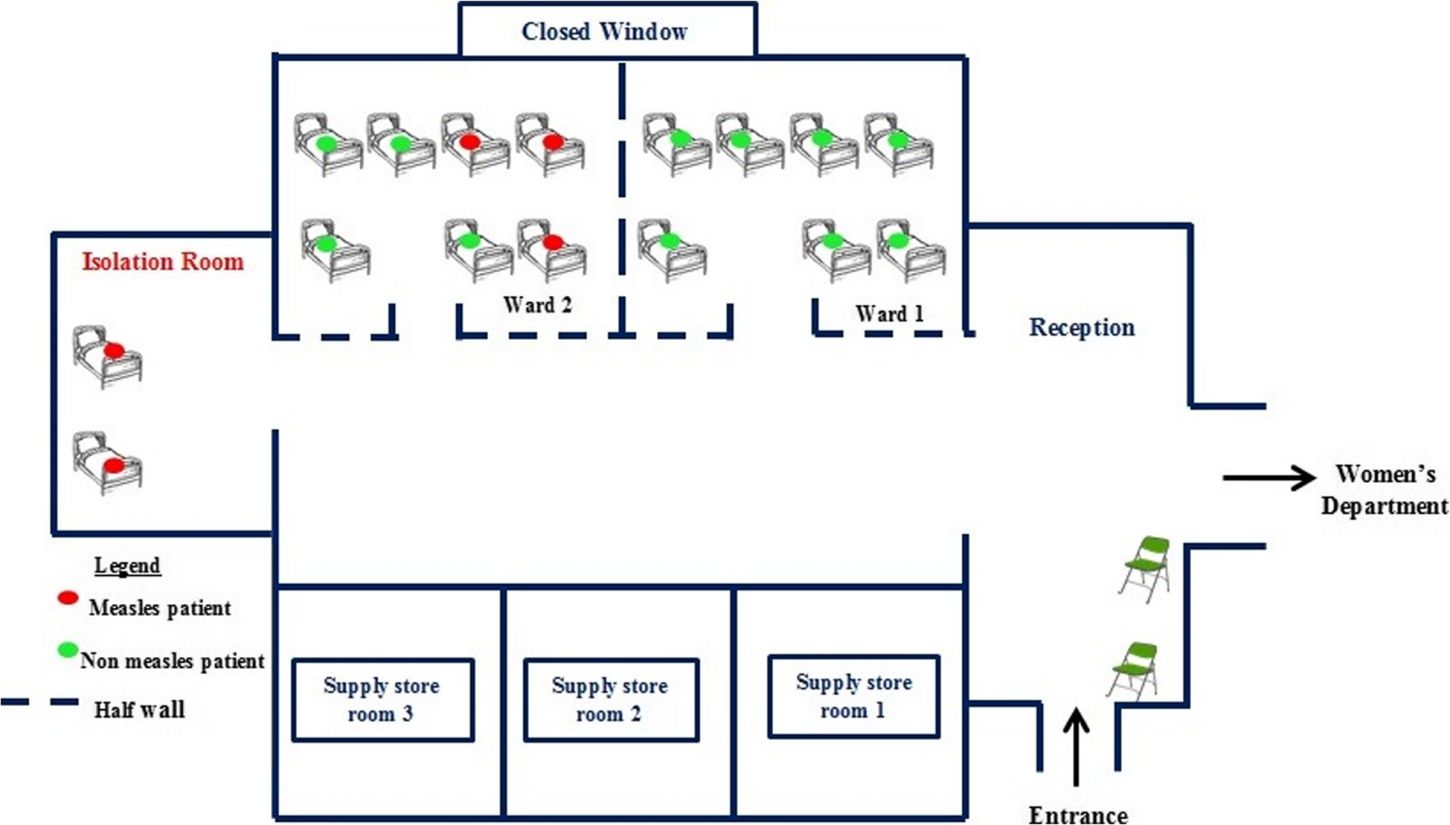
Layout of the pediatric department at Lyantonde Hospital during a measles outbreak: Lyantonde District, Uganda, June–September 2017

The pediatric department at Lyantonde Hospital had 2 wards. Initially, measles patients and other patients were mixed in the same wards because the measles diagnoses had not been made. After the measles outbreak was confirmed based on results from Uganda Virus Research Institute, the hospital attempted to put non-measles patients into Ward 1 and suspected measles patients into Ward 2. However, the 2 wards were adjacent to each other and only separated by a half-constructed wall; air moved freely between the 2 wards. Moreover, when Ward 1 exceeded its capacity, some non-measles patients were transferred into Ward 2. Windows of both wards were kept closed. Later during the outbreak, a windowless “isolation room” was set up to hold critical measles patients. The “isolation room” was at the extreme end of the pediatric department; patients had to pass through the whole department to access this room, allowing measles and non-measles patients to mix (Fig. 3). During our environmental assessment, we observed free mixing of measles and non-measles patients in the reception area.

## Discussion

This measles outbreak was facilitated by mixing of measles and non-measles patients when they were hospitalized in the pediatric department of Lyantonde Hospital. As in previous studies, the role of uncontrolled nosocomial transmission of measles in the propagation of community outbreaks cannot be refuted [11–13]. In this study on-site assessment of the pediatric department revealed infection-control lapses, this likely facilitated measles transmission. As non-measles patients were infected from the measles patients and developed symptoms, they returned to seek care at the pediatric department and transmitted the disease to other non-measles patients, creating vicious cycles of transmission. The subsequent public health interventions, e.g., moving the “isolation” room outside of the pediatric department and the emergency mass vaccination campaign might have helped to break the vicious cycles.

Measles viruses become airborne after patients expel the droplets by coughing or sneezing. These droplets may remain suspended in the air and remain infectious for up to 2 h after the infectious patient has departed the area [12]. Transmission occurs when susceptible individuals share the same confined spaces with, or within 2 h of the departure of infectious patients [13]. However since patients can become infectious as early as four days before the classic measles rash appears, infection can spread for days before the need for isolation becomes apparent, making healthcare facilities a fertile ground for measles transmission [14–20].

Preventing nosocomial transmission should be an important part of the overall measles control strategies [13]. When a non-immune person is exposed to measles virus, a prophylactic measles vaccination could provide protection if administered within 72 h of exposure [12, 21]. Therefore, researchers have advocated for pediatric departments to offer measles vaccination to pediatric patients during active measles outbreaks to prevent nosocomial transmission, especially in pediatric healthcare settings [22]. In addition WHO recommends that a supplementary dose of measles vaccine is given to infants from 6 months of age during a measles outbreak as part of intensified service delivery [23]. However, these approaches were not used during this outbreak.

Measles is endemic in Uganda. Outbreaks have been recently reported in various parts of the country, and this outbreak is likely linked to one of more of those recent outbreaks, several of which have also been linked to exposure in healthcare settings [14]. In another outbreak, measles was associated with congregation of children at water-collection points [24]. In this outbreak, visiting water-collection points was protective. This might be because going to the communal water-collection point was a sign of being healthy and healthy children would have less chance of going to and being exposed at the pediatric department of Lyantonde Hospital.

In this study history of Measles vaccination was protective of measles virus infection. Measles vaccination is the best strategy for preventing measles outbreaks and achieving l measles elimination [9]. WHO recommends a 2-dose vaccination administered at 9 and 15–18 months of age, and a VC at ≥80% for the 2-dose vaccination schedule [9].

The estimated VE for the one-dose vaccination during this investigation (95%) was higher than previous estimates of 64% [14] 75% [24] and 77% based on a review of published literature on the field effectiveness of live attenuated measles containing vaccines [25]; however, the confidence interval for the current estimate (80–100%) overlaps with those of the previous estimates [17–85% [14] and 25–92% [24], respectively]. Observed VE in the field varies and is influenced by many factors, such as number of doses administered, vaccine quality, cold-chain failure, and host factors [26].

In addition to ensuring a high VE, maintaining a high VC through routine vaccination activities is crucial for measles control. WHO-AFRO has set a target of ≥90% in VC to achieve herd immunity for preventing measles outbreaks [5]. In this investigation, the overall estimated VC for all persons was 76%, the age-specific VC estimates were ranging from 72 to 83% and the administrative VC estimate was 83%. All these were well below the WHO-AFRO target of ≥90%. The low VC likely facilitated the current outbreak. Recent investigations in Uganda [14, 24] and in other countries [14, 27, 28] have also attributed measles outbreaks to low VC. These low VC estimates showed weaknesses in the routine vaccination system.

### Limitations

This investigation had multiple limitations. Due to the need of providing a quick answer for outbreak control, we only included 34 early cases in the case-control study, which might have severely limited the power of the study to find exposure risk factors other than the most overwhelming ones. Moreover, the exposure risk factors of the early cases might have been different from the ones during the later stage of the outbreak. We used controls in the case-control study to estimate VC. By using this method, we assumed that the controls were representative of the general population. This assumption might not be valid, thereby introducing bias in the VC estimate. However, the VC estimated from the case-control study (76%) was close to the administrative VC (83%); the latter is known to often overestimate the true VC. These data suggested that the bias, if any, might have not been substantial. The vaccination status of some of the children was based on their parents’ recall, which might have been inaccurate.

## Conclusions

We conclude that this measles outbreak was amplified through nosocomial transmission in the pediatric department in Lyantonde Hospital. At our recommendation, Lyantonde Hospital moved the isolation ward to a room outside of the building, which did not share air with the other buildings of the hospital. The hospital also triaged suspected measles patients (with fever and rash) and isolated them in this properly-constructed new separated room.

Subsequent to the outbreak, the district health authorities conducted training of the village health teams on signs and symptoms of measles, and on the appropriate process to promptly report suspected measles cases. In addition, the district health office changed their policy from conducting vaccinations only on certain fixed days each month to providing vaccination on any day a child was brought to the health facilities. After these intervention measures were implemented, the outbreak declined and eventually stopped.

## Data Availability

The datasets used and analyzed during the current study are available from the corresponding author on reasonable request.

## Abbreviations

UMoH: Uganda Ministry of Health
VC: Vaccination coverage
VE: Vaccine effectiveness
